# Case Report: An Infant with Severe Thrombocytopenia Diagnosed with Type 2B von Willebrand Disease Due To a De Novo p.Val1316Met Mutation

**DOI:** 10.4274/tjh.galenos.2020.2020.0213

**Published:** 2020-11-19

**Authors:** Junjie Fan, Jing Ling, Huifeng Zhou, Jie He, Shaoyan Hu

**Affiliations:** 1 Children’s Hospital of Soochow University, Department of Hematology and Oncology, Jiangsu, China

**Keywords:** Type 2B von Willebrand disease, VWF gene, Thrombocytopenia

## To the Editor,

Type 2B von Willebrand disease (vWD 2B) is a rare disease that is difficult to diagnose. It usually presents with a normal or decreased platelet count with bleeding tendency. Clinically, it can be confused with immune thrombocytopenic purpura (ITP), which results in improper treatment. Herein, we report a case of an infant with severe thrombocytopenia diagnosed with vWD 2B.

The female infant was found to have some petechiae on her body when she was 6 days old. A routine blood test in a local hospital showed a decreased platelet count of 19x10^3^/µL, a low hemoglobin level of 100 g/L, and a normal white blood cell count of 12.1x10^3^/µL. Serum bilirubin was elevated to 19 mg/dL. Cranial ultrasound showed subependymal hemorrhage and bilateral intraventricular hemorrhage. After treatment with platelet transfusion, intravenous immunoglobulin, methylprednisolone, and vitamin K1, the baby’s bilirubin and hemoglobin normalized and her platelet count increased to 80x10^3^/µL initially, but it dropped down to approximately 20x10^3^/µL thereafter.

At the age of 41 days, the infant was transferred to our hospital. On admission, the physical examination was unremarkable except for some ecchymosis on the body. Her family history included no family members with low platelet levels or with bleeding diathesis. Laboratory evaluations were notable for severe thrombocytopenia (platelet count: 16x10^3^/µL) but otherwise normal hemoglobin and white blood cell count values. The mean platelet volume was 13.4 fL (normal range: 7.4-11 fL). A peripheral blood smear revealed nonspecific thrombocytopenia without platelet clumping. The antinuclear antibody investigation was negative, and the prothrombin time, activated partial thromboplastin time, and antithrombin activity were all normal, as well. Bone marrow aspiration revealed  abundant megakaryocytes with dysmaturity. A presumptive diagnosis of ITP was made, and intravenous immunoglobulin (1 g/kg, single dose) and dexamethasone (0.6 mg/kg once a day for 4 consecutive days) were administered to the infant. The platelet count reached 54x10^3^/µL after treatment, the ecchymosis on the body disappeared, and she was discharged from the hospital. During the 6 months following discharge, she received no further treatment, and her platelet count was approximately 40x10^3^/µL.

**Figure 1:**
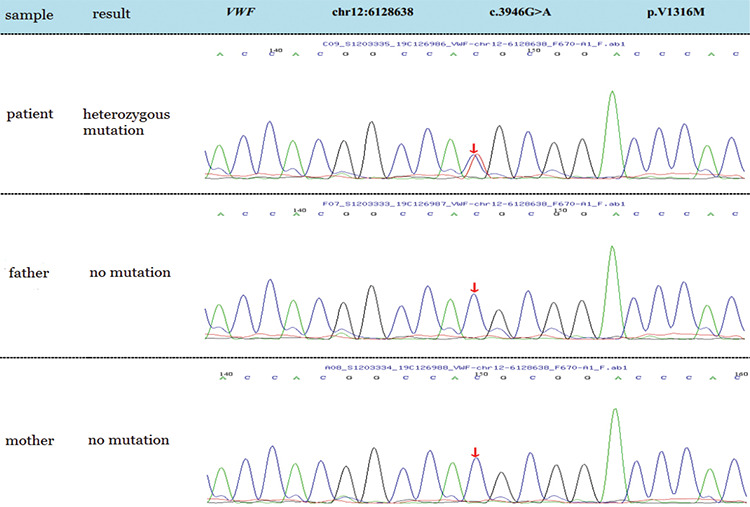
A heterozygous c.3946G>A mutation occurred in this patient, whose parents did not have the same mutation by Sanger sequencing.

During her hospitalization, DNA analysis by next-generation sequencing (NGS) was performed, and the results were received half a month after her discharge. The results revealed a heterozygous *VWF* c.3946G>A, p.Val1316Met mutation, with replacement of valine by methionine at position 1316 (dbSNP: rs61749397). This gene variant was checked for the baby’s parents by Sanger sequencing and it proved to be a de novo mutation (Figure 1). According to the guidelines of the American College of Medical Genetics and Genomics, it is a pathogenic variant, and it is one of multiple reported causative gene variants. Thus, the diagnosis of type 2B vWD was definite in this case. We suggested further tests for VWF:RCo and VWF:Ag, but the baby’s parents considered the gene sequencing results sufficient for the diagnosis and were unwilling to authorize any other laboratory tests.

Von Willebrand disease (vWD) is a common inherited bleeding disorder and is classified into three types: type 1, type 2 (2A, 2B, 2M, and 2N), and type 3. The type 2B vWD subtype is a rare autosomal-dominant transmitted variant of vWD accounting for fewer than 3% of all vWD cases. It is characterized by structural or functional defects in von Willebrand factor (vWF). A gain-of-function mutation in the A1 domain causes spontaneous binding of vWF to GP1bα receptors on platelets, causing increased clearance of vWF multimers and platelets, and, additionally, abnormal vWF can modify megakaryocytopoiesis, both of which result in thrombocytopenia [1,2]. vWD 2B is the only type of vWD recognized that may cause thrombocytopenia, and clinically, it can easily be confused with ITP. Traditional test findings such as enhanced ristocetin-induced platelet aggregation or the absence of high-molecular-weight multimers can support the diagnosis of this disorder; however, as it is a disease caused by gene mutation, genetic testing is straightforward and can confirm the diagnosis [3]. vWD 2B patients present with moderate-to-severe lifelong bleeding. The bleeding risk is higher in those with thrombocytopenia than in those without [4]. The severity of thrombocytopenia is variable and may be related to the type of mutation [5]. Moreover, pathologic stress situations such as infection, pregnancy, and surgery can increase the level of mutated vWF and the clearance of platelets, leading to lower platelet counts [4]. The mainstay of vWD 2B treatment is vWF replacement. Plasma-derived vWF/FVIII concentrates including Humate-P work by introducing normal vWF into the circulation [6]. Desmopressin is not considered a therapeutic option because it causes the release of stored abnormal vWF, which can worsen thrombocytopenia [7]. In the case of extremely low platelet counts, platelet transfusions may be necessary, even though transfusion has only transient efficacy [8].

The likelihood that this infant would have had ITP or alloimmune thrombocytopenia at the same time was low. The outcome of this patient’s bone marrow smear was consistent with the diagnosis of ITP; however, no clinical or laboratory parameters were sufficient to establish this diagnosis accurately. The diagnosis of ITP is one of exclusion. In the current case, the onset of thrombocytopenia was too early (6 days after birth) for the immune system to produce anti-platelet autoantibodies, and additionally, her platelet count did not normalize after treatment with intravenous immunoglobulin and dexamethasone; thus, we excluded the diagnosis of ITP. Alloimmune thrombocytopenia can occur very early after birth, but it is a self-limiting disorder that usually resolves within 2-4 weeks [9]. For this infant, her thrombocytopenia lasted more than half a year, demonstrating that alloimmune antibodies were not the cause of her thrombocytopenia, at least after the neonatal period. 

It is very rare for a patient with vWD 2B to develop symptoms in the early neonatal period of severe thrombocytopenia and intracranial hemorrhage. The diagnosis could have been missed if NGS had not been completed. NGS has revolutionized the study of genomics and molecular biology throughout the last decade. An increasing number of diseases are being definitely diagnosed using NGS techniques. Thus, we recommend NGS testing for refractory thrombocytopenia patients to determine whether any hereditary disorders such as vWF 2B exist, even in those without a family history of similar complaints or known bleeding disorders.
